# Learning the Abstract General Task Structure in a Rapidly Changing Task Content

**DOI:** 10.5334/joc.176

**Published:** 2021-07-07

**Authors:** Maayan Pereg, Danielle Harpaz, Katrina Sabah, Mattan S. Ben-Shachar, Inbar Amir, Gesine Dreisbach, Nachshon Meiran

**Affiliations:** 1 Department of Psychology and Zlotowski Center for Neuroscience, Ben-Gurion University of the Negev, Beer-Sheva, Israel, 8410501; 2 Department of Psychology, University of Regensburg, Regensburg, Germany

**Keywords:** Rapid Instructed Task Learning, multilevel modeling, instructions-based performance, prospective memory

## Abstract

The ability to learn abstract generalized structures of tasks is crucial for humans to adapt to changing environments and novel tasks. In a series of five experiments, we investigated this ability using a Rapid Instructed Task Learning paradigm (RITL) comprising short miniblocks, each involving two novel stimulus-response rules. Each miniblock included (a) instructions for the novel stimulus-response rules, (b) a NEXT phase involving a constant (familiar) intervening task (0–5 trials), (c) execution of the newly instructed rules (2 trials). The results show that including a NEXT phase (and hence, a prospective memory demand) led to relatively more robust abstract learning as indicated by increasingly faster responses with experiment progress. Multilevel modeling suggests that the prospective memory demand was just another aspect of the abstract task structure which has been learned.

## 1. Introduction

Consider valet parking, where the valet driver is required to drive and park newly arriving cars without assistance. Did this driver develop car-specific expertise, i.e. did she become an expert in driving each and every car type? It is more probable (and parsimonious when considering processing resources) that she learned the general structure for operating different types of cars, a structure which could be described as a set of “slots” or placeholders for casting the changing aspects. These aspects which change as a function of the specific car type include the location of the hand break, the location of the light knob, size of the vehicle, etc. Such an abstract structure operates like a syntax in that it enables quick adaptation to a completely new car model, by simply filling the placeholders with information. In this study we examined what characterizes such abstract learning through performance benefits seen in the concrete but constantly changing tasks, which share a common abstract structure. Below we outline the theoretical and empirical background which has led our hypothesis.

The ability to apply learning from one task to other tasks while generalizing common task properties has been discussed in the literature for decades. In 1980, Thorndyke and Yekovich defined *schema* as an abstraction that encodes conjoint properties of a typical instance of an event, object or situation. According to these authors, schema learning is pivotal for reasoning and for making predictions. Abelson (1981) focused on one form of schema: the knowledge of a stereotypical sequence of events, which was conceptualized as a *script*. Schema and scripts enable people to form expectations and make inferences from relatively minimal information, and eventually are needed for designing a behavior compatible for the given circumstances. This body of literature relies heavily on text comprehension tasks (e.g., Abbott et al., 1985; Zacks & Tversky, 2001), a fact which possibly limits its wider applicability.

Miller argued that in order to practice an intended behavior and match it appropriately to experience, one needs a cognitive system that is complex enough to acquire the “rules of the game” (Miller, 2000). It has long been suggested that in humans and possibly also in other sophisticated organisms such as apes, the pre-frontal cortex takes a critical role in this process (Miller, 1999). Particularly, Miller claimed that rule learning depends on associating two pieces of information such as when we learn that “red light” means “stop”. Instead of learning rules from scratch, humans can flexibly rely on previously learned simple rules and combine them to meet the demands of a complex novel task (Cole et al., 2011). This ability, demonstrated in Cole et al.’s work, presumably implies an abstract coding on some level, as the basic rules learned in one context can be applied in broader novel contexts.

Cole et al. (2011) used a paradigm that demands participants to exploit Rapid Instructed Task Learning (RITL) in order to quickly adapt to a new task relying on mere instructions. This type of paradigm, in which the tasks are constantly changing, is suitable for the research of abstract representations. This is so because the specific concrete rules become irrelevant once they had been executed and a new task began, thus ruling out the contribution of associative retrieval of a *concrete* rule in any performance improvement that may be observed in the course of the experiment. Moreover, the fact that participants cannot rely on long-term memory of Stimulus-Response (S-R) mapping rules is supported by previous studies suggesting that working memory (rather than long-term memory) plays an important part in RITL performance (Meiran et al., 2012, 2016; Pereg & Meiran, 2019).

It should be noted that this type of learning has already been indicated in different fields, such as artificial grammar learning (Lieberman et al., 2004), where participants learn a grammatical structure in an unconscious schematic learning; as well as causal models (Kemp et al., 2010), where an abstract representation of the causal schema was termed ‘learning to learn’, following Harlow (1949). A similar construct, ‘structural learning’, relates to learning the general form of rules that control the abstract task structure. ‘Structural learning’ stands in contrast to ‘parametric learning’ which deals with learning the specific S-R mapping controlling the current task (Braun et al., 2010).

Interestingly, similar capacities have been exemplified in animals as well, demonstrating neocortical schemas in rats that serve as mental frameworks for implementation of novel information (Tse et al., 2007). Another classic animal study indicated abstract task structure learning (or ‘learning sets’) with monkeys, who showed performance improvement throughout a series of discrimination tasks, despite the change in task-content (Harlow, 1949).

However, not many studies addressed abstract structure learning specifically in speeded tasks. Among the few studies that addressed this question is Badre et al.’s (2010) who presented participants with a hierarchical (second-order) abstract rule (relative to “flat”, first-order one-to-one S-R mapping rules) and showed that this arrangement resulted in higher learning-related performance gains (see also Kayser & D’Esposito, 2013). Moreover, Collins and Frank (2013) formulated a model that describes how a learner might infer a latent task set structure, showing that cognitive control is applied in order to learn abstract task sets. However, that study used a reinforcement learning task, where the flat task-rules had to be inferred as well (in addition to the abstract task set). In the current study, we aimed to explore a related yet different phenomenon. Specifically, our focus could be viewed as being related to skill learning (in a rule-based task), where the newly learned skill needs to be extended beyond specific contexts.

**Figure 1:**
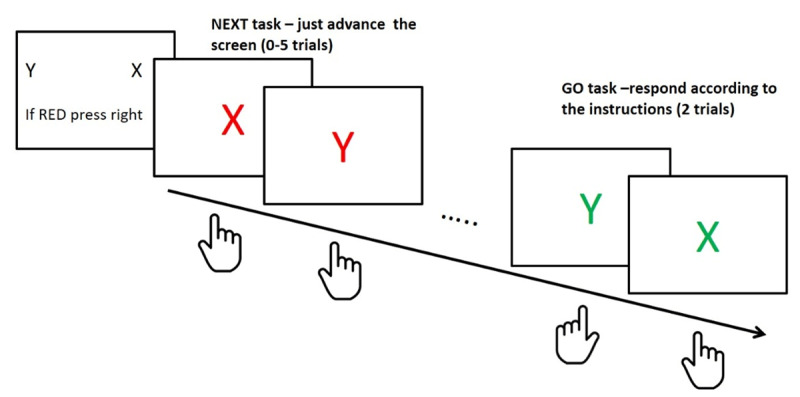
Trial sequence in the NEXT paradigm. Each mini-block consisted of two novel stimulus-response mapping rules (e.g., X-RIGHT and Y-LEFT). On each mini-block, participants are first instructed towards performance in the GO task, in which the stimuli appear in green color and only performed twice. After the instructions and prior to the GO task, a number of targets in red color require a fixed NEXT response (right/left, counterbalanced between participants and constant throughout the experiment).

To sum up, in the current study, we explored learning of an abstract structure in a RITL choice reaction task, the NEXT paradigm (described in ***Figure 1*** and in detail in the Method section 2.2 below; Meiran et al., 2015). In this task, participants encounter a series of simple tasks, each comprising a novel set of two S-R mapping rules. We predicted that there would be a training effect (i.e., quicker responses with experimental progress) despite the fact that the stimuli, and accordingly S-R mapping rules, in each mini-block were novel and have never been trained beforehand (Badre et al., 2010; Harlow, 1949).

Our aim was to understand the process of abstract task learning. To do so, we used multilevel models in order to examine whether learning parameters vary in different experimental conditions, reflecting distinct learning requirements. Since these analyses partly pool together different experiments that were conducted during this study, in the following section we give a brief overview that skims through the experiments, describing the rationale for each experiment, as well as descriptive results and some basic inferential statistics.

### 1.1. Experimental Overview

The study involved five experiments. In Experiment 1, we show that participants’ performance is improved during the course of the GO task of the NEXT paradigm (***Figure 1***), despite the fact that the first-order task-rules are constantly changing. Experiment 2 showed that this result did not replicate when the NEXT task was omitted from the experiment. In Experiment 3 we manipulated the presence/absence of a NEXT task and show that performance improvement is significantly greater when the NEXT task is included in the experiment as compared to when it is not included. Finally, in Experiment 4, we show that this effect likely stems from the need to deal with a prospective memory task, and not from a more specific response-conflict. The rationale for running the experiments is elaborated below. Experiment 5 was added at a later stage and is described in detail after the modelling results.

## 2. Methods for Experiments 1–4

### 2.1. Participants

In Experiment 1, 175 Hebrew speaking participants (29 females, mean age = 22.71, SD = 2.39) took part in a cognitive training experiment (see Pereg, Shahar, & Meiran, 2019, for a full report of the study). Here, we report the pre-training performance in the NEXT paradigm.

In Experiment 2, 100 Hebrew speaking Ben Gurion University students participated in the experiment (74 females, mean age = 23.45, SD = 1.66), for course credit or for monetary compensation (25 NIS, ~7$ U.S dollars). Participants were randomly assigned to each of five conditions (elaborated below) and reported having normal or corrected-to-normal vision, including intact color vision, and not having diagnosed attention deficits.

In Experiment 3, 40 participants (35 females, mean age = 22.93, SD = 1.43) similar to those in Experiment 2, were randomly assigned to one of two conditions. Finally, Experiment 4 involved 40 Regensburg University (German) students (35 females, mean age = 21.85, SD = 2.0), who participated in return for course credit or monetary compensation (7 Euros, ~8$ U.S dollars). One participant was excluded due to an especially high proportion of errors (PE = 0.52).

### 2.2. Materials and procedure

In Experiment 1, the task was the same task used in Meiran et al., (2015). It consisted of 55 mini-blocks, each instructing a novel mapping towards the GO task, relating two stimuli (that constantly changed across the mini-blocks) to the right/left keys (the “L” key and the “A” key, respectively, covered with stickers). In each mini-block, participants were asked to study the mapping appearing in white and afterwards to press the spacebar (but not sooner than after 3 seconds had elapsed) and advance to the following phase. In the NEXT task, the stimulus appeared in red, and participants were asked to press a fixed key to advance to the next screen. The number of NEXT task trials in a mini-block (0–5) was drawn from a pseudo-exponential distribution (10%, 30%, 20%, 20%, 10%, and 10% for 0–5 NEXT trials respectively). In the GO task, the participants needed to react according to the primary mapping for merely two trials after which they received response times (RT) and accuracy feedback regarding their GO performance in the current mini block. In these two GO trials, one of the two new stimuli was drawn randomly with replacement. Following the feedback screen, a new mini-block began with the instruction of two new rules.

The stimuli for the NEXT paradigm were chosen from a pool of 220 stimuli, made of 24 Hebrew letters, 10 digits, 26 English letters, 20 symbols (e.g., arithmetic symbols, drawn from Microsoft PowerPoint symbol tool), and 140 pictures (e.g., objects and shapes, drawn from free internet image search bases). The stimuli were presented at the size of 3 × 3 cm. The stimuli on each mini-block were drawn from the same stimulus category (e.g., two Hebrew letters, two symbols, etc.), and each stimulus was only used once throughout the experiment. The NEXT paradigm was programmed in E-Prime 2.0 (Psychology Software Tools, 2010).

In Experiment 2, participants were randomly assigned to one of five conditions and the experimenter was blind to the condition participants were assigned to. The five conditions are described in Supplementary Materials Online. Generally, the paradigm was similar to the NEXT paradigm described in ***Figure 1*** (with 118 mini-blocks), except for omitting the NEXT task and the three seconds minimum for advancing the instructions screen. Other modifications which were made are described below. The experiment was programmed in OpenSesame (Mathôt et al., 2012).

Experiment 3 involved two conditions: one equivalent to Experiment 1 and one equivalent to the parallel condition in Experiment 2. In other words, the two conditions differed only with respect to whether a NEXT task was included. Finally, in Experiment 4, the stimuli were the same as those in Experiment 1 except for the omission of the Hebrew letters (given that the participants were German). These letters were replaced by familiar icons (e.g., the Microsoft Word ™ icon). Twenty participants conducted the standard NEXT task in which participants employed one of the two keys that were also used in the GO task to advance the screen (as in all the other experiments) and twenty participants used the spacebar instead.

### 2.3. Data analysis

Our main focus in this study was the GO task. More specifically, we focused on the first GO trial in each mini-block, which is the only trial that could be considered purely instructions-based (since more advanced trials could already be based on learning from experience occurring during the first GO trial (Meiran et al., 2015; Pereg et al., 2019; Pereg & Meiran, 2019). All erroneous trials were omitted from the RT analysis, as well as trials with RT under 100 ms or over 4000 ms.

## 3. Experiments’ Rationale and Results

### 3.1. Experiment 1

**Figure 2:**
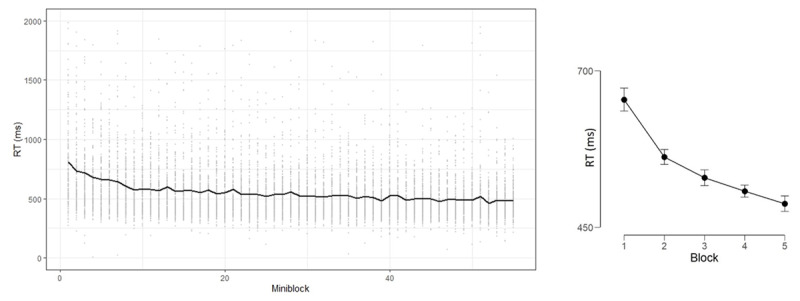
Left panel: RT as function of Miniblock in Experiment 1. Grey dots illustrate the individual variance around the mean. Right panel: RT as a function of Block, error bars represent 95% Bayesian credible interval.

Experiment 1 examined learning in the first GO trials during the course of the NEXT paradigm (Meiran et al., 2015). This was accomplished by re-analyzing existing data reported in some published papers (Pereg et al., 2019; Shahar et al., 2018). As hypothesized, the results show better performance (shorter RT) with experiment progress (***Figure 2***, left panel).

In order to allow basic statistical inference, we binned the mini-blocks into five Blocks comprising 11 trials each (***Figure 2***, right panel). We performed an Analysis of Variance (ANOVA) and Bayesian ANOVA (Rouder et al., 2012), to estimate the relative odds of H1 and H0 given the data (assuming equal priors for H0 and H1, and using the default H1 priors). We further report BF_10_, the relative odds of H1 and H0, BF_10_ > 3 is considered a reliable effect, and BF_10_ < 0.33 allows accepting the null hypothesis (Jeffreys, 1961). The B/ANOVA demonstrated a robust Block effect [F(4,696) = 95.11, p < .0001, η_p_^2^ = 0.35, BF_10_ = 8.93e^+60^].

### 3.2. Experiment 2

Experiment 2 was aimed to study which mechanism allows for such abstract learning, given the constant change in S-R mapping rules. Therefore, we designed a few conditions such that comparing between them would have allowed us to isolate the specific process that is crucial for generalized learning to occur *if it occurs*. Since we were interested in the learning effect of the GO task, the NEXT task was completely omitted, meaning that participants always executed the S-R mapping twice immediately after receiving the mapping instructions. The conditions in this experiment included a conceptual replication condition and four additional conditions that served as control conditions.

**Figure 3:**
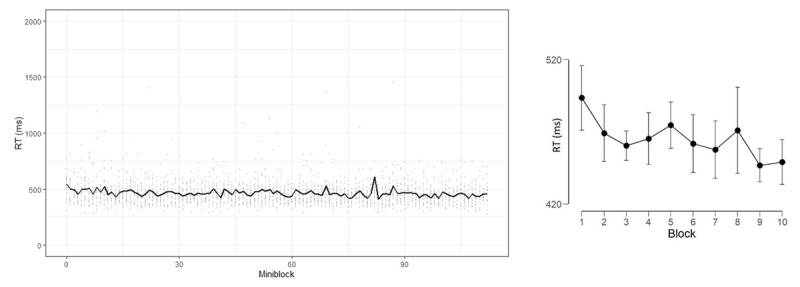
Left panel: RT as function of miniblock in the conceptual replication condition in Experiment 2. Individual data are shown in dots, and the mean can be seen in the line. Right panel: RT as a function of Block, error bars represent 95% Bayesian credible interval.

The conceptual replication condition was similar to the NEXT paradigm, such that each mini-block involved two novel S-R mapping rules, though without a preceding NEXT task. There were four additional conditions which were designed to help isolate the locus of the learning effect, *if found*. Given that there was no evidence for learning, and that the control conditions were designed to identify the locus of training – these conditions became somewhat useless. For this reason, the rationale for designing the control conditions as well as the related results are presented in Supplementary Materials Online. Here, we will focus on the conceptual replication condition. ***Figure 3*** (left panel) shows the descriptive results of this condition, which surprisingly did *not* show robust evidence for learning. As in Experiment 1, we divided the mini-blocks into Blocks. Since the experiment was longer, this resulted in 10 Blocks (this was done in order to maintain the number of mini-blocks per Block similar across experiments). The ANOVA showed a significant effect for Block, which was not supported by the BANOVA [F(9,171) = 2.19, p < .05, η_p_^2^ = 0.10, BF_10_ = 1.17], pointing rather to an indecisive result (Jeffreys, 1961).

Descriptively, it seems that performance in the first miniblocks was considerably better (quicker RT) relative to Experiment 1, which could explain the surprising toned-down learning during the experiment, such that there was less to learn to get to an asymptote level. As we see it, the most important finding in this experiment with respect to our question is the *lack* of replication of Experiment 1’s results, suggesting that the inclusion of the NEXT task (which was not included in Experiment 2) had a significant contribution to the observed learning.

In retrospect, it appears as if the NEXT paradigm should be conceived as resembling a prospective memory task (Brandimonte et al., 2014), and this has turned out to be a critical aspect for abstract learning to take place. The term “prospective memory” describes remembering activities that should take place in the future and are referred to as delayed intentions. Being able to successfully execute such tasks was found to challenge attentional resources (Smith, 2003), as well as working memory (Smith & Bayen, 2005), at least in some contexts (though see Anderson et al., 2018; and Heathcote et al., 2015 for a different interpretation).

Within the current study, the prospective memory (NEXT) task structure was originally introduced in order to measure the automatic effects of instructions (Meiran et al., 2015, 2017; Pereg & Meiran, 2020). Nonetheless, the results of Experiment 2 demonstrate the importance of this component for learning in the GO task, and thus were tested in the following experiments.

### 3.3. Experiment 3

Experiment 3 was conducted in order to replicate the difference between Experiment 1 and the conceptual replication condition in Experiment 2, while controlling for potential software differences and participants characteristics (see Methods section). Therefore, the only difference between the two experimental conditions would be the involvement of the prospective NEXT task. This experiment involved two conditions -with/out a NEXT task.

**Figure 4:**
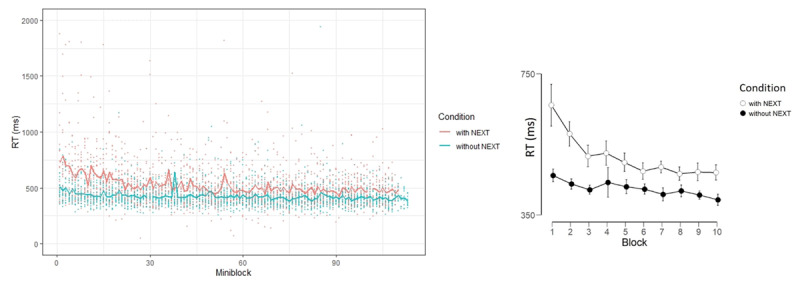
Left panel: RT as function of miniblock in Experiment 3, different conditions are marked with different colors. Individual data are shown in dots, and the mean per condition can be seen in the lines. Right panel: RT as a function of Block and Condition, error bars represent 95% Bayesian credible interval.

In this experiment, we predicted that the results would show that significant learning occurred in the condition including a NEXT task (replicating Experiment 1), and no learning in the without-NEXT condition (replicating Experiment 2). Descriptively, the results seem similar to those found in the previous experiments. We divided the mini-blocks into 10 Blocks, and the B/ANOVA showed a robust Block effect [F(9,342) = 20.04, p < .001, η_p_^2^ = 0.34, BF_10_ = 5.72e^+20^] and a robust interaction between Block and Condition [F(9,342) = 6.78, p < .001, η_p_^2^ = 0.15, BF_10_ = 1.86e^+6^]. Given this robust interaction, we tested the learning effect in each condition separately and surprisingly found a robust learning effect in both conditions [F(9,171) = 17.19, p < .001, η_p_^2^ = 0.47, BF_10_ = 9.00e^+16^ (with-NEXT); F(9,171) = 4.27, p < .001, η_p_^2^ = 0.18, BF_10_ = 392.08 (without-NEXT)]; though it was significantly larger in the with-NEXT condition, suggesting that the prospective component contributed to the *size* of the learning effect but did not dictate its presence (see ***Figure 4***). Moreover, the results do *not* tell what exact aspect about the presence of a NEXT task contributed to the occurrence of learning.

In the original NEXT paradigm, there is a potential conflict between the NEXT and GO responses, such that in some NEXT trials, the NEXT response conflicted with the response that would have been generated on the basis of the newly instructed rules and in other trials there was no such conflict (Meiran et al., 2017). Hence, it it conceivable that resources had to be allocated for resolving the response conflict. Additionally, resources might have been allocated for early selection which could have prevented response conflict, or at least could alleviate it. For example, paying greater attention to the red/green color of the stimulus that indicated the task (NEXT vs. GO) in the miniblock. Accordingly, it is possible that the increased learning effect reflects the learning to overcome conflict. An alternative hypothesis would be that a more general prospective memory process is responsible for the increased learning effect. Experiment 4 was meant to try to clarify this issue.

### 3.4. Experiment 4

The goal of this experiment was to test whether the critical aspect for the increased learning effect is the presence of a (potential) conflict in the NEXT task. If this is the case, the learning effect is predicted to abolish once the possibility of a response conflict is removed by having an ongoing (NEXT) task without a response conflict. On the other hand, if a more general prospective memory component is responsible for this effect, then the prediction is that the effect would remain similar even if the NEXT response does not create a potential response conflict. To test this issue, we compared a condition similar to the with-NEXT paradigm employed thus far, except for using a non-lateralized NEXT response (pressing the spacebar with both hands) to prevent the response conflict.

**Figure 5:**
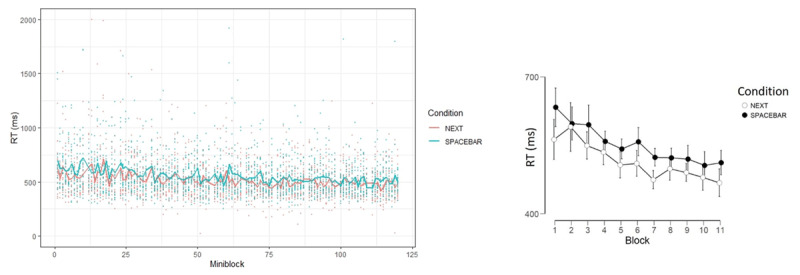
Left panel: RT as function of miniblock in Experiment 4, different conditions are marked with different colors. Individual data are shown in dots, and the mean per condition can be seen in the lines. Right panel: RT as a function of Block and Condition, error bars represent 95% Bayesian credible interval.

We predicted that if the critical component for a robust learning effect is the involvement of potential response conflict in the NEXT task, then a greater learning effect should be observed in the condition where the NEXT response was similar to the previous experiments (involving a lateralized response). However, if the more general prospective component is critical, the results should be similar in both conditions and roughly replicate the preceding experiments. The results seem to support the latter hypothesis. We divided the mini-blocks into 11 Blocks, and the B/ANOVA showed a robust Block effect [F(10,380) = 14.16, p < .001, η_p_^2^ = 0.27, BF_10_ = 3.64e^+18^] without an interaction between the conditions, indicating decisive support for the null hypothesis (equivalence) [F(10,380) = 0.66, p = .76, η_p_^2^ = 0.2, BF_10_ = 0.02] (see ***Figure 5***).

In this experiment we found evidence *against* the hypothesis that the potential response conflict in the NEXT task is the locus of the increased learning effect (i.e., that better dealing with the conflict is being learned during the experiment). Instead, the results suggest that what is being learned is dealing with the prospective-memory component of the task, at least in the current methodological framework. Other alternative explanations are proposed in the General Discussion.

### 3.5. Interim Discussion

To sum up the results thus far, abstract learning seems possible in a novel two-choice task where the task involves a prospective memory component. However, much less learning or even no learning at all was observed otherwise, possibly since the task demands were too light. Nonetheless, we currently cannot reach decisive conclusions regarding the nature of learning. Many possibilities remain, including that without the NEXT task, learning was obscured by the easier conditions, or that a different learning process took place in both conditions. Furthermore, our conclusions remain limited given that thus far, statistical inference was based on averaged data across blocks, and that it was not based on an explicit learning model. To address these limitations, we turned to mixed-modeling, containing both fixed and random effects. Such an approach would possibly allow us to account for the *individual* variance and extract specific learning parameters in the different conditions.

## 4. Modeling

In order to test whether different learning processes took place in the different conditions, we first aimed to select the most suitable learning model for the task at hand. According to the power law (Logan, 1992):

RT = \left({y0 - yf} \right){P^{ - {\rm{exp}}\left(\alpha \right)}} + yf

Where *RT* is the reaction time, *P* is the amount of practice received (or the number of mini-blocks in our task), *yf* is the asymptote that indicates the limit of performance, α is the learning rate and *(y0-yf)* is the difference between initial (*y0*) and final performance (*yf*) (Logan, 1992); i.e., the learning rate is highest at the beginning of training, and it becomes gradually slower as training proceeds.

The power law of practice was considered ubiquitous for speeded tasks. However, it was later suggested that relative to averaged data, in individual fitting, there is not enough evidence that the power function provides better fit than an exponential decay function (Heathcote et al., 2000). Eventually, Heathcote et al. demonstrated that an exponential decay function provides a better fit when considering individual-level data. Thus, we turned to understand learning by fitting Heathcote et al.’s model:

RT = \left({y0 - yf} \right){e^{ - \alpha P}} + yf

For the purpose of modeling, we pooled participants across experiments to maximize statistical power. Specifically, for the “with NEXT” condition, we pooled Experiment 1, and the “with NEXT” conditions from Experiments 3 and 4. The “without NEXT” condition was formed by pooling the respective condition from Experiments 2 and 3.

We adopted a model-comparison approach, whereby the exponential decay function would be compared with other models. First, the linear model was selected as the null model, assuming a constant learning rate throughout the experiment. Second, we opted to test whether a mixed model (involving individual differences in parameter values) would have an advantage over a fixed model (without these individual differences). We also designed several models, such that each model involves a different set of free parameters. Comparing between these models allowed us to test the necessity of each free parameter.

### 4.1. Results

Since Experiment 1 involved a smaller number of mini-blocks relative to Experiments 3 and 4 (55 vs. 110 mini-blocks), the analyses were performed on the lower common denominator of 55 mini-blocks. We note that it seems that the most (or all) learning took place at the beginning of the experiment, and thus, this cutoff only trims the asymptotic part of the function (see ***Figures 4*** and ***5***). In addition, given the theoretical and empirical importance of the first trial described above, the models focused on the first GO trial, as did the previous analyses.

The null baseline models were fixed-linear and mixed-linear. A fixed linear model estimates one slope coefficient, whereas a mixed-linear model also estimates individual linear slopes. For the exponential decay, we used the SSAsymp function in R (Pinheiro & Bates, 2006) which estimated the logarithm of the learning rate *a* instead of estimating *a* directly:

RT = \left({y0 - yf} \right){e^{ - \exp \left({{\rm{log}}\left(\alpha \right)} \right)P}} + c

This function is a self-starting asymptotic regression function that guesses its own starting parameter values. Thus, it solves a convergence problem that is otherwise created. SSasymp was used combined with *nls*, the standard R function for fitting non-linear trends (Nash, 2014).

The models were planned to allow a comparison between different assumptions in order to assess the different learning processes between the conditions. This required to directly test whether there is a difference between the conditions (with and without NEXT) within a model that is fit to the entire dataset.

***Table 1*** summarizes the initial models that tested whether the experimental conditions differ in terms of the parameters which characterize their learning. This was done by comparing a baseline model in which the conditions were pooled (i.e., assuming the same function for the conditions) to models allowing the conditions to differ in terms of their learning parameters (a “Condition” free parameter). To allow this examination, we tested each model at least twice, once with and once without a Condition free parameter; and then compared the models in terms of the Bayesian Information Criterion (BIC; lower values suggest better model fit; Schwarz, 1978).

{\rm{BIC}} = - 2{\rm{logLikelihood}} + {\rm{log }}\left({\rm{N}} \right){\rm{*P}}

**Table 1:** BIC values for fixed (no individual differences)/mixed (individual differences) models of a linear/non-linear function.


# MODEL	MODEL DEFINITION	ARE EXPERIMENTAL CONDITIONS ALLOWED TO DIFFER IN TERMS OF PARAMETER VALUES	BIC

1	Fixed linear	no	172,514.4
	
2		yes	172,198.8

3	Mixed linear	no	168,962.0
	
4		yes	168,786.0

5	Fixed non-linear	no	172,374.1
	
6		SP+A+LR ^a^	171,989.7
	
7		SP	172,011.4
	
8		A	172,151.0
	
9		LR	172,007.9
	
10		SP+A	171,981.2
	
11		SP+LR	172,016.7
	
12		A+LR	171,983.7

13	Mixed non-linear ^b^	no	168,265.3
	
14		SP+A+LR	168,275.0
	
**15**		**SP**	**168,242.3**
	
16		A	168,282.7
	
17		LR	*Did not converge ^c^*
	
18		SP+A	168,264.4
	
19		SP+LR	168,263.8
	
20		A+LR	*Did not converge*


^a^ SP = Starting point; A = asymptote; LR = learning rate. ^b^ For simplicity, in this set of models, the random effect was estimated for all three parameters. This will be further tested for the best fitting model. ^c^ Models 17 and 20 did not converge after exceeding the number of maximal iterations, (which was set to 10,000), suggesting that these models are unsuitable for the data.

Note that adding free parameters (such as when allowing conditions to differ in a parameter) is *penalized* in the BIC value, and thus if a model is preferred *albeit* estimating an extra free parameter, this suggests that the conditions robustly differ. BIC differences of 6 points or more are considered to be substantial.

Models 1–4 assumed that performance improvement is described by a linear function. They differ in whether there are individual differences in the parameters of the model (“mixed”) and whether the conditions differ. The comparison between the four models shows that learning is better described by exponential decay, and that individual differences in model parameters are substantial. Models 5–12 all assume that learning is described by exponential decay, but none of them allows for individual differences in the model’s parameters (they are all “fixed”). Models 5–12 differ from one another in terms of whether the conditions differ and in which parameter. Models 5–12 were outperformed by Models 13–20 that allow for individual differences in all the three parameters of the exponential-decay model. Thus, the real comparison should be made between the (more realistic) models which allow for individual differences in the learning parameters. Models 13–20 each represents a difference between the conditions in a set of learning parameters. Clearly, Model 15 outperformed all other models in this category.

To sum up the results thus far, the best model was Model 15, a mixed non-linear model, assuming random effects (individual differences) in all of the three parameters of the learning function, and allowing conditions to differ only in their starting point parameter. The second-best model in this category was Model 19, assuming that the conditions differ in both the starting point and the learning rate. The ΔBIC between two models is translated into an approximate Bayes Factor (Neath & Cavanaugh, 2012), such that BF = e^0.5*ΔBIC^. The ΔBIC between Model 15 and Model 19 was 21.5, making the BF_10_ = 46,630.03, which is considered as decisive evidence in favor of Model 15 (Jeffreys, 1961). To explore which individual differences must be assumed, we examined Models 21–26. All these models assume that the conditions differ in SP, but each of them represents a different combination of individual differences (see ***Table 2***). This examination shows that Model 15, assuming that all learning parameters show individual differences (“have random effects”), is indeed the best model for the data.

**Table 2:** BIC values for models comparing different random effects in the mixed non-linear model.


# MODEL	MODEL DEFINITION	RANDOM EFFECTS	BIC

**15**	Mixed non-linear, SP differs between the experimental conditions	**SP+A+LR**	**168,242.3**
	
21		SP	168,823.7
	
22		A	169,392.7
	
23		LR	*Did not converge*
	
24		SP+A	168,375.8
	
25		SP+LR	*Did not converge*
	
26		A+LR	168,545.7


Taken together, the results can be summarized as follows: (1) The exponential-decay function showed best fit to the data (relative to a linear function); (2) the difference between the experimental conditions (i.e., with/out a prospective component, the NEXT task) influenced only the SP, i.e., the *starting point* of the exponential decay, and did neither influence the asymptote nor the learning rate; this was seen in the fact that Model 15 outperformed Models 14, 17, 19, and 20 (all assuming condition differences in the learning rate) and Models 14, 16, 18, 20 (all assuming condition differences in asymptote); and (3) the mixed model showed better fit than the fixed model, supporting robust *individual differences* in all the three parameters of the learning function. These points will be elaborated in the General Discussion.

The behavioral and modeling results seem to suggest that the NEXT phase, with the added prospective memory component, increases the total to-be-learned. This difference between the conditions in terms of total to-be-learned might reflect the increased working-memory and attentional demands that may be associated with prospective memory such as monitoring the task for the appearance of green stimuli, indicating the transition to the task-execution (GO) phase (Smith, 2003; Smith & Bayen, 2005). This account faces a challenge because, in addition to the prospective-memory demand, the NEXT task adds a delay between the instructions and their execution during the GO task.

Moreover, the with/out NEXT task conditions differ with respect to the type of the task-switch taking place when transitioning to the GO task. Specifically, when NEXT is present, the task switch is between two tasks (NEXT and GO). In contrast, when a NEXT task is absent, the GO task comes after the instructions screen. To deal with the delay issue, and to some extent also with the type-of-switch issue, we ran Experiment 5.

## 5. Experiment 5

**Figure 6:**
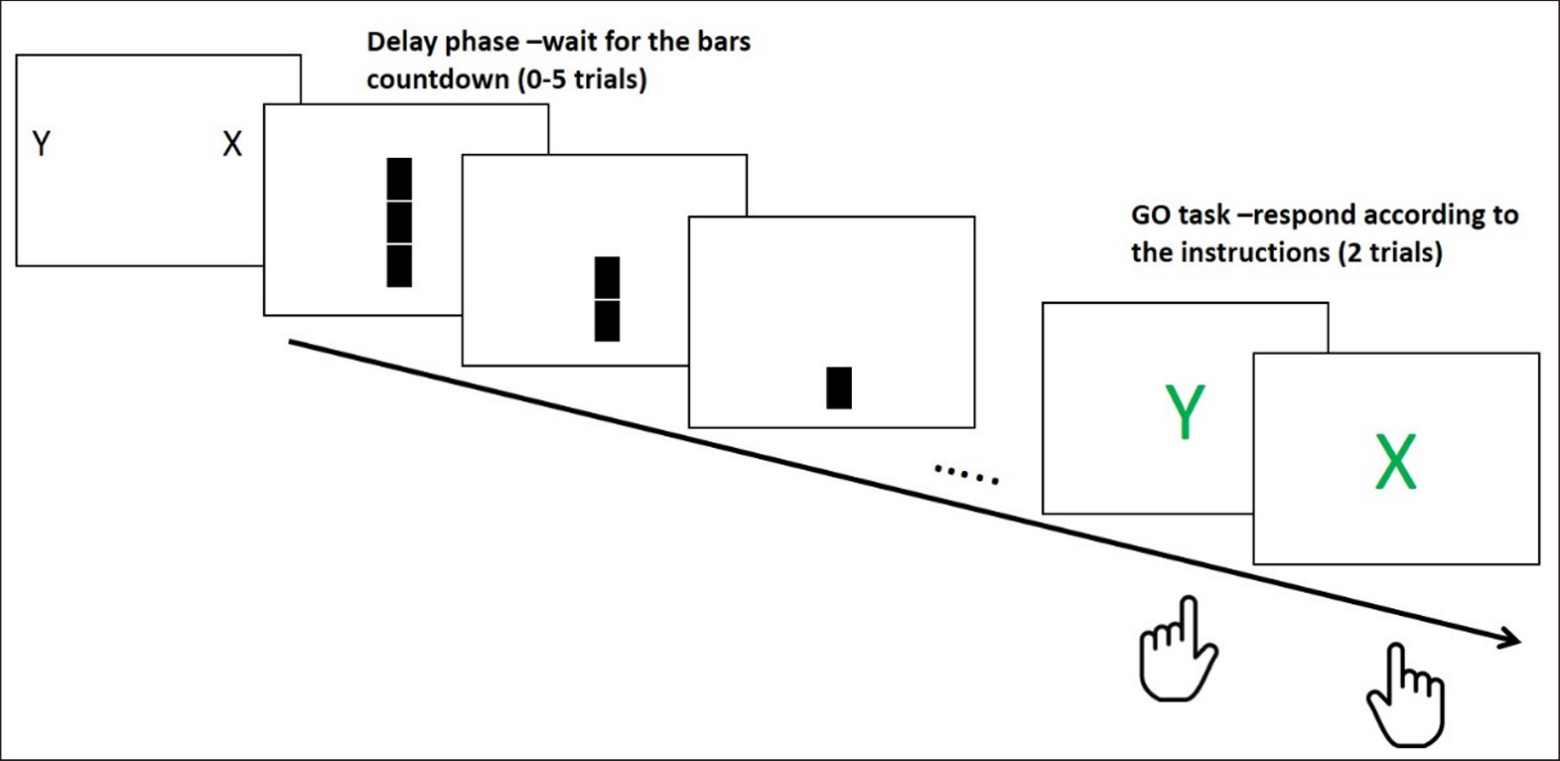
Trial sequence in the “without NEXT_delay” condition.

In Experiment 5, we compared the “without NEXT” condition, to another “without NEXT” condition in which a delay period was introduced between the instructions and GO performance (see ***Figure 6***). This “without NEXT_Delay” condition thus closely mimics the timing of the with-NEXT condition, though without the need to respond in a secondary (NEXT) task. If the delay created by the NEXT task as well as the transition from the NEXT task to the GO task is the critical difference between the with/out NEXT conditions, then the without NEXT_delay condition should show initial poor performance and a very gradual learning as the with-NEXT condition did in the previous experiments.

### 5.1. Method

#### 5.1.1. Participants

Forty-one participants (31 females, mean age = 23.39, SD = 1.03), with similar characteristics to participants from Experiments 2 and 3, were randomly assigned to the “without NEXT_immediate” or “without_NEXT_delay” condition. Participants received course credit for their participation in the experiment.

#### 5.2.2. Materials and procedure

The experiment closely resembled Experiment 3. Due to COVID-19, the experiment was held online, but actions were taken to make the procedure as similar as possible to that of the previous experiments. Participants performed the experiment at home, using their computers with software written in OpenSesame-Web 3.3.6 (Mathôt et al., 2012) and exported to JATOS server (*https://www.jatos.org/*). After registering to the experiment, participants were invited to a Zoom (*https://zoom.us/*) video meeting with the experimenter. During this video conversation, the experimenter explained about the experiment and sent the participant the JATOS link for the experiment. The Zoom meeting was kept in the background throughout the experiment, in order to make sure that participants did not encounter any technical problems. Importantly, the sound and camera of both the participants and the experimenter were turned off during the experiment and were used only in the case of technical problems (which did not occur for any of the participants in this experiment).

The “without NEXT_immdeiate” was a close replication of the “without NEXT” condition, only with 55 mini-blocks. The “without NEXT_delay” condition closely followed the “with NEXT” condition, only that instead of NEXT trials, we added delay trials. In those trials, a vertical bar appeared, whose length represented the delay, and shrank at a constant rate so that when it disappeared, the GO task started. The length of delay phase followed that of the NEXT task (Experiments 1 and 3), such that its length was pseudo-exponentially determined to be between 0 and 5 seconds. Each bar was presented for 1000 ms and participants were instructed to wait for the GO task to begin, and then execute the instructions. In this experiment, there was no break between the practice and the actual experiment, and the first three mini-blocks (the number of practice trials in the previous experiments) were considered as practice and were omitted from the analyses.

### 5.2. Results

**Figure 7:**
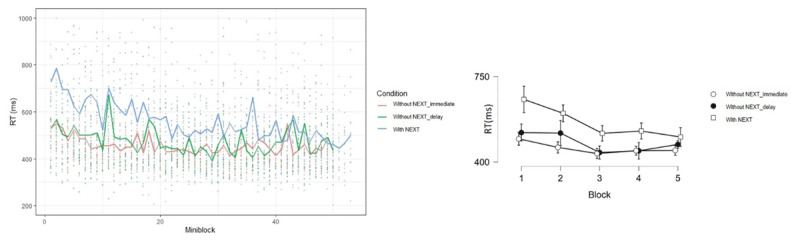
Left panel: RT as function of mini-block in Experiments 3 (with NEXT condition) and 5 (without NEXT conditions), different conditions are marked with different colors. Individual data are shown in dots, and the mean per condition can be seen in the lines. Right panel: RT as a function of Block and Condition, error bars represent 95% Bayesian credible interval.

We reasoned that the prospective component of the NEXT phase (and not the delayed GO phase) is the critical factor for the difference between the with/out NEXT conditions. Accordingly, the prediction was for a lack of difference in the learning rate between the two groups, and for very little learning in both of them. Descriptively, the immediate and delayed “without NEXT” conditions seem to show very little learning throughout the task. Like in the previous experiments, we binned the mini-blocks into Blocks (containing 11 mini-blocks each, as in all the preceding experiments, see ***Figure 7***). In order to help the readers compare the current results to the “with NEXT” condition, we added the results of the “with NEXT” condition from Experiment 3 to ***Figure 7***.

Focusing on Experiment 5, the B/ANOVA showed a robust Block effect [F(4,156) = 12.90, p < .001, η_p_^2^ = 0.25, BF_10_ = 1.87e+6], indicating learning, but no interaction between Block and Condition [F(4,156) = 1.52, p = .20, η_p_^2^ = 0.04, BF_10_ = 0.27; with BF permitting to accept the null hypothesis (Jeffreys, 1961)]. This result shows that some learning took place in both conditions, but its rate was statistically equivalent across the two groups. To compare the results from the current experiment to those from the “with NEXT” condition from Experiment 3, we performed an additional B/ANOVA, with Experiment as an independent variable. The results demonstrated a robust interaction between Experiment and Block [F(4,236) = 4.30, p < .01, η_p_^2^ = 0.07, BF_10_ = 7.53]. Taken together, the results suggest that the “without NEXT” conditions in this experiment significantly differ from the “with NEXT” condition from Experiment 3, but that the two groups in this experiment did not differ from each other.

To sum up, Experiment 5 rules out delay as the critical factor and thus supports the notion that the prospective component of the NEXT task as being a major contributor to abstract learning. The experiment does not completely rule out type-of-switch as an alternative account, but it makes it less likely to be correct. Specifically, the two groups in the present experiment had very different types of switch to the GO task: from instructions vs. from monitoring the bar yet produced statistically equivalent results. Additional theoretical implications are discussed in the General Discussion.

## 6. General Discussion

In this study, we tested whether abstract task learning is feasible in a RITL procedure, where the task-sets (each comprising two S-R associations) constantly change. We hypothesized that transfer from one task to the other would be considered abstract once the concrete task elements (the stimuli linked to the right-left keys) constantly change. To examine this issue, we performed a series of five experiments that were designed to specifically target the essential task element that allows for such abstract learning to take place. Surprisingly, the results show that abstract learning was most robust when the task involved a prospective-memory element, i.e. when the newly learned rules had to first be kept in memory until completing the execution (as opposed to mere waiting) of another (fixed, NEXT) task. To further examine this observation, we employed mixed modeling that demonstrated that learning was best described by an exponential decay function that suggests a constant learning rate that is applied to a gradually reducing total to-be learned, similar to that seen in other cognitive tasks (Heathcote et al., 2000; Logan, 1992). In addition, the models demonstrate that the prospective memory component only altered the starting point; i.e., the prospective-memory-related difference could be attributed to the amount of information that had (or could be) learned.

When observing ***Figure 4***, for example, the conclusions drawn on the basis of modeling may not seem intuitive, since the drop in RT is much sharper with NEXT than without NEXT. This is exactly where modeling helps. Specifically, according to the exponential decay model, the total to-be-learned is the difference between SP (starting point) and A (asymptote). Given equal asymptotic RT in the two conditions, the difference in SP implies more to-be-learned with NEXT. Additionally, according to the model, the total to-be-learned is multiplied by a fraction that becomes increasingly small with learning progression (P). Thus, the reduction in RT as a result of learning is proportional, and when the proportion is the same, greater absolute reduction will be seen with greater to-be-learned, i.e., when NEXT is present.

Nonetheless, we note that learning to deal with a prospective memory component throughout task performance could be considered abstract learning in its own right, given that it does not involve specific S-R mapping rules. Our modeling results support this possibility to some degree by showing that this aspect is indeed learned, such that the difference between the experimental conditions (with and without a prospective memory component) reflects the additional learning required to manage this added process. Experiment 5 further clarified the importance of the added process, by demonstrating that the difference does not simply stem from the delay between the instructions and GO execution, which is less abstract in this sense. Another alternative account that can be ruled out concerns temporal expectancy and task-switching. In all the with-NEXT conditions, the first GO trial came at an unexpected time and involved a switch from NEXT to GO, whereas the transition to the GO trial was temporally expected in the without-NEXT condition, and the switch was from a response-less task (instructions, or monitoring the diminishing bar) to the GO task. However, if temporal expectancy and switching from NEXT to GO were the critical factor, we would expect that the 2^nd^ GO trial in the with-NEXT condition would behave like the without-NEXT condition, but this did not happen (see Supplementary Materials). In fact, the results from the (expected) 2^nd^ GO trial closely resemble those from the (less expected) 1^st^ GO trial.

One hypothesis to explain the current results is “structural learning” mentioned earlier (Braun et al., 2010), which could possibly be achieved by building a mental representation of the task with two empty right/left “slots” or “placeholders” for setting the two new stimuli such that the only thing left to be learned in each mini-block is which stimulus fills which slot. However, while this hypothesis might be sufficient to explain the (quick) learning in the “without NEXT” conditions, it does not explain why learning was more robust in the with-NEXT condition. To explain it, we refer to the “task buffer” hypothesis, in which instructions are hypothesized to temporarily be on hold so that they are shielded against interference until when implementation is required (Cole et al., 2017; see also Dreisbach, 2012). According to this idea, when the NEXT task is present (delaying implementation and generating interference), it encourages relying on abstract task representations, which in turn enable relatively far transfer of learning precisely due to the abstract nature of the learned representation. Accordingly, Dreisbach and Wenke (2011), suggested that abstract learning (high-order as opposed to S-R mapping rules) supports transfer to different contexts. Further support for these notions is found in Sabah et al.’s works (Sabah et al., 2019, 2020), showing that when relatively more abstract learning is required (by means of hindering concrete learning), greater transfer gains are observed.

The current study also has important methodological implications for RITL research. Specifically, studies focusing on (or incrementing on) RITL research often use a series of novel tasks to facilitate task-set learning processes in each of the new tasks (Cole et al., 2013; Liefooghe et al., 2012; Ruge & Wolfensteller, 2010). However, the results of the current study suggest that the nature of the general task structure changes gradually via abstract learning processes.

In addition, it is important to keep in mind that the modeling indicated mixed effects in all the parameters describing the learning process, suggesting individual differences in this type of learning ability. This finding stresses the need to consider individual differences in future RITL research. Moreover, the results also stress the need to come up with RITL-related experimental paradigms that are better suited to bypass abstract learning, perhaps by constantly changing the task-structure. Finally, the current results suggest that caution should be exercised when generalizing across studies with and without a prospective memory component (Liefooghe et al., 2012; Meiran et al., 2015; vs. Ruge & Wolfensteller, 2010), since this aspect seems to change the nature of the task to some degree.

In summary, the current study exemplifies abstract task learning in a choice reaction task in which the concrete task elements are constantly changing, such that any learning that took place during the experimental session was attributed to hierarchically higher task elements. The study thus shows that “learning to learn” can be found even with rapid learning in speeded tasks.

## Data Accessibility Statement

Raw data can be found at OSF (*https://osf.io/jh8rd/*).

## Additional File

The additional file for this article can be found as follows:

10.5334/joc.176.s1 Supplementary Materials. 1: Experiment 2; 2: Second GO trial.
