# Supplementary material for: Learning the Abstract General Task Structure in a Rapidly Changing Task Content
Source: J Cogn. 2021 Jul 7;4(1):31. doi: 10.5334/joc.176 (PMC8269791; doi:10.5334/joc.176)
Supplement: Supplementary Materials. — 1: Experiment 2; 2: Second GO trial. [file joc-4-1-176-s1.pdf]

Supplementary Materials 1 – Experiment 2

As has been mentioned in the main text, Experiment 2 involved the main conceptual replication condition, which was similar to that of Experiment 1 except for omitting the NEXT phase and four control conditions. When designing these conditions, we (tentatively) adopted a modified version of Pashler and Baylis's (1991) model as a heuristic. This means that we are not committed to the assumptions, and have just used them as a heuristic. Specifically, we assumed the following processing stages: stimulus detection (presumably facilitated by having a fixed stimulus position that eases fixating), stimulus encoding, S-R rule retrieval, and response preparation. We further assumed that, given the commonly held view that long-term memory represents stable statistical regularities, elements that remain stable throughout the experiment can be learned, while those that constantly change cannot be learned. Finally, we assumed that learning involves the most concrete elements possible. For example, in the NEXT paradigm, there is an abstract task structure involving the instructions format, mini-block structure etc. More concrete elements include specific S-R mapping rules, for instance.

The main (conceptual replication, "all novel" condition) condition was hypothesized to involve a few elements that could be learned/improve: (1) target stimulus position was constant and could facilitate *stimulus detection* processes; (2) *stimulus encoding* processes; and (3) *S-R rule retrieval* were required throughout the experiment since the S-R rules changed; (4) *response preparation*; and (5) *rule switching* were all necessary processes throughout the task, and therefore we hypothesized that they would improve, at least in an abstract manner. For example, since different stimuli were involved in each mini-block, the stimulus encoding process could not be considered a concrete-repeating element, and the *abstract* encoding process could have enhanced through improvement in the encoding strategy. Finally (6) the *abstract task-structure*

presumably involved instructions with two “place-holders” for novel stimuli. This could be conceived as the mapping involving two generic rules: (1) IF {} THEN {press right}, and (2) IF {} THEN {press left]. We refer to the abstract task-structure as the most abstract element that could be learned during task performance. The second (“two novel”) condition involved switching between two fixed S-R mapping rules that were novel only at the beginning of the experiment, when they were first instructed. This condition was hypothesized to involve the same processes as the first condition, with the addition of concrete S-R rules, thus supposedly easing the *stimulus encoding* and the *rule-retrieval* processes. In addition, the task structure in this condition was made more concrete, since participants can employ the two specific S-R mapping rules. The third (“one novel”) condition involved one fixed S-R mapping rule, which was novel only when it was first instructed at the beginning of the experiment. Thus, the *rule switching* process was omitted (made easy) for this condition. In the fourth and fifth conditions, we completely omitted the need to hold a newly instructed rule in WM and thus eased *stimulus encoding* and *S-R rule retrieval.* We did so by applying familiar, non-arbitrary and compatible S-R rules. Specifically, the stimuli were left/right pointing arrows to point to left/right responses. Similar to the third condition which involved one S-R mapping rule with two stimuli, the fourth (“two arrows”) condition involved switching between the two arrows during the execution phase, and thus stimulus encoding and response selection were still required. The fifth and final (“one arrow”) condition involved showing two arrows during the instructions phase, but only one of the arrows appeared during the execution phase, making the *response preparation* much easier, and even redundant once the participants have learned that only one arrow appears throughout the experiment.

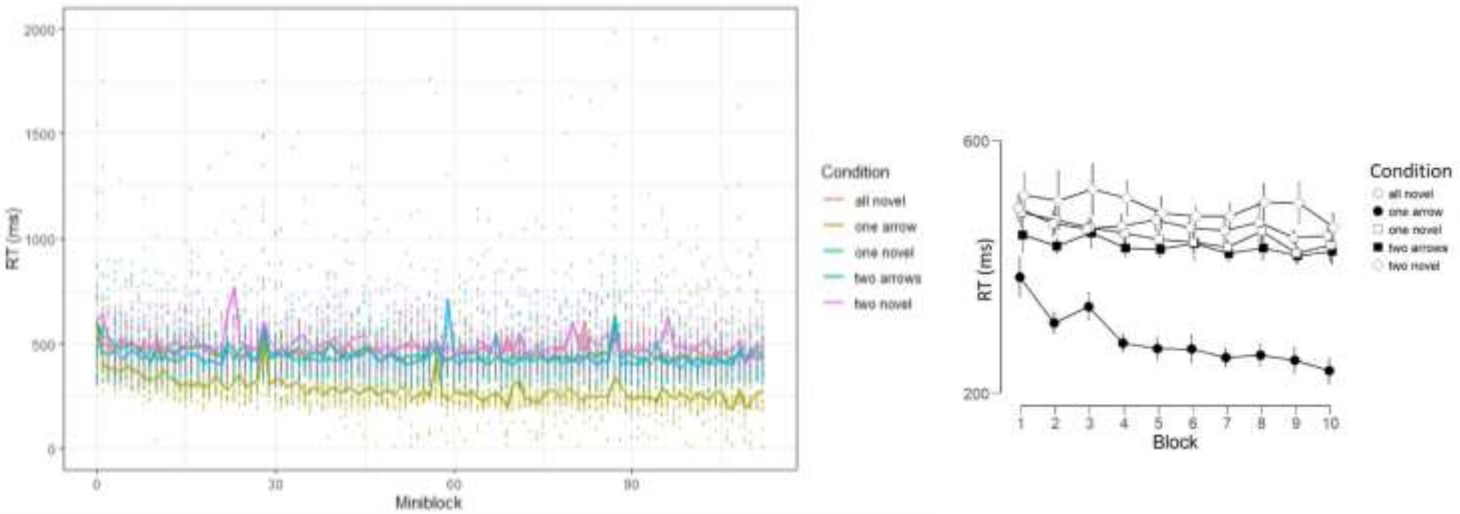

Figure S1. *Left panel: RT as function of miniblock in Experiment 2, different conditions are marked with different colors. Individual data are shown in dots, and the mean per condition can be seen in the lines. Right panel: RT as a function of Block and Condition, error bars represent 95% Bayesian credible interval.*

Figure S1 (left panel) shows the descriptive results and demonstrates that the fifth “one arrow” condition was at odds with the other four conditions. As in Experiment 1, we divided the mini-blocks into Blocks. Since the experiment was longer, this resulted in 10 Blocks (this was done in order to maintain the number of mini-blocks per Block similar across experiments). The B/ANOVA demonstrated a robust effect for Block [$F(9,819)=17.38$, $p<.001$, $\eta_p^2=0.16$, $BF_{10}=2.39e^{+19}$] and an interaction between Block and Condition [$F(36,819)=2.45$, $p<.001$, $\eta_p^2=0.10$, $BF_{10}=4.40e^{+2}$]. Surprisingly, there was no robust evidence for learning in the conceptual replication (“all novel”) condition [$BF_{10}=1.17$, which is considered an indecisive result (Jeffreys, 1961)].

There was robust evidence for learning in merely two conditions: the “one arrow” condition ($BF_{10}=1.00e^{+21}$), and surprisingly - the “one novel” condition ($BF_{10}=6{,}280.19$). The learning in the “one arrow” condition was not predicted. In retrospect, it might reflect the

participants' learning that only one of the arrows (either pointing left or right) appears during the experiment. If this is the case, participants can perform during this condition as in a "simple RT" task, where response selection is completely redundant. Therefore, we decided to not further analyze this condition, as it may reflect a different learning process than intended. As for the "one novel" condition, this condition was expected to involve very low WM demands (due to the repetition of one arbitrary rule) and no rule-switching, and thus no abstract task-structure was expected to be learned. The common factor for both conditions is the lack of rule-switching demands, but this is also true for the "two arrows" condition, which did not demonstrate learning [BF_{10}=0.29, allowing accepting the null hypothesis]. Thus, it is currently unclear why this condition produced a robust learning effect.

Given that we chose to focus on comparing abstract learning with/out the involvement on the NEXT phase in an "all novel" NEXT paradigm, these control conditions are not further discussed in the study.

Supplementary Materials 2 - Second GO trial

Although the first GO trial serves as the purest measure for instructions-based learning (given that the second GO trial already benefits from any learning that took place during the execution of the first GO trial); for completeness sake, we also report the results of the second GO trial. Another motivation for this set of analyses concerns the fact that unlike the first GO trial that came at a relatively unexpected point in time and involved a task-switch (from NEXT to GO), the 2nd GO trial could be fully temporally expected and did not involve a task-switch.

As in the main text, for each experiment we illustrate the descriptive pattern, and report the B/ANOVA results for Block progression, and interactions with the experimental condition, where applicable.

Experiment 1

The B/ANOVA demonstrated a robust Block (learning) effect [$F(4,696)=110.291$, $p<.001$, $\eta_p^2=0.39$, $BF_{10}=1.41e^{+69}$] (see Figure S2).

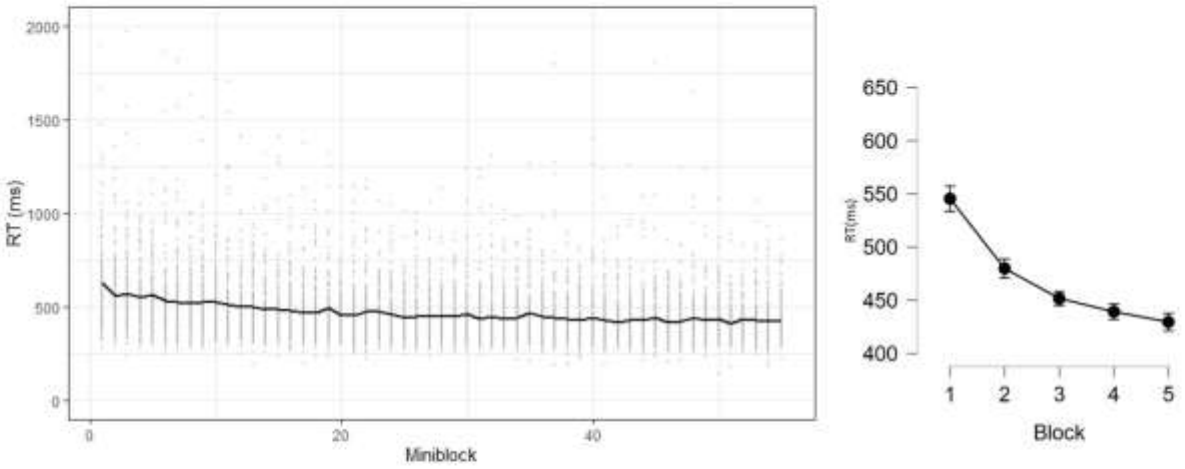

Figure S2. *Left panel: RT in Trial 2 as function of Miniblock in Experiment 1. Grey dots illustrate the individual variance around the mean. Right panel: RT as a function of Block, error bars represent 95% Bayesian credible interval.*

Experiment 2 (conceptual replication condition)

As in Experiment 1, and unlike in Trial 1, the B/ANOVA showed a surprising significant effect for Block [$F(9,171)=5.87$, $p<.001$, $\eta_p^2=0.24$, $BF_{10}=36,786.39$]. That is, unlike the results

concerning the first trial, the second trial demonstrated pronounced learning (Figure S3). Note however, that this result is the opposite from what would be predicted based on the hypothesis that what has been learned was dealing with task-switching/ temporal unexpectedness.

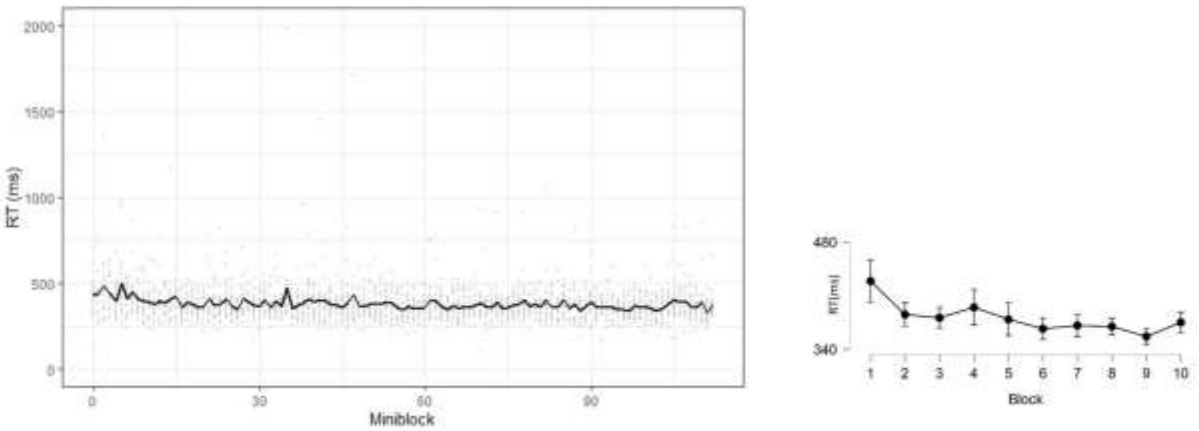

Figure S3. *Left panel: RT in Trial 2 as function of Mini-block in Experiment 2. Grey dots illustrate the individual variance around the mean. Right panel: RT as a function of Block, error bars represent 95% Bayesian credible interval.*

Experiment 3

the B/ANOVA showed a robust Block main effect [$F(9,342)=15.79$, $p<.001$, $\eta_p^2=0.29$, $BF_{10}=3.45e^{+15}$] and an interaction between Block and Condition $F(9,342)=7.81$, $p<.001$, $\eta_p^2=0.17$, $BF_{10}=5.59e^{+7}$] (see Figure S4), whose BF indicates a decisive result, one echoing the parallel interaction in the 1st GO trial.

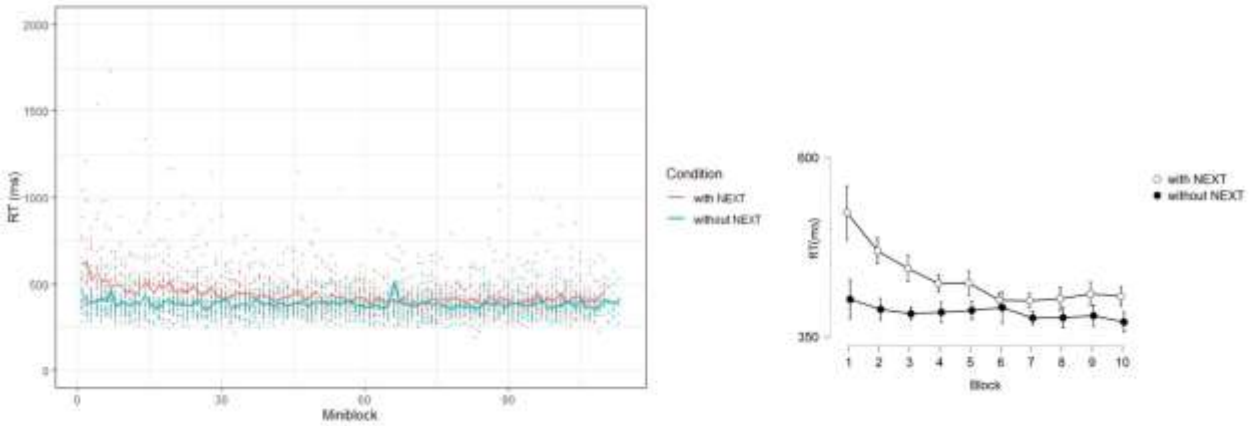

Figure S4. *Left panel: RT in Trial 2 as function of miniblock in Experiment 3, different conditions are marked with different colors. Individual data are shown in dots, and the mean per condition can be seen in the lines. Right panel: RT as a function of Block and Condition, error bars represent 95% Bayesian credible interval*.

Experiment 4

The B/ANOVA showed a robust Block effect [F(10,380)=9.90, p<.001, η_p^2=0.21, BF_{10}=4.38e^{+11}] with an indecisive interaction between Block and Condition, with BF close to allowing acceptance of the null hypothesis [F(10,380)=1.79, p=.06, η_p^2=0.04, BF_{10}=0.42].

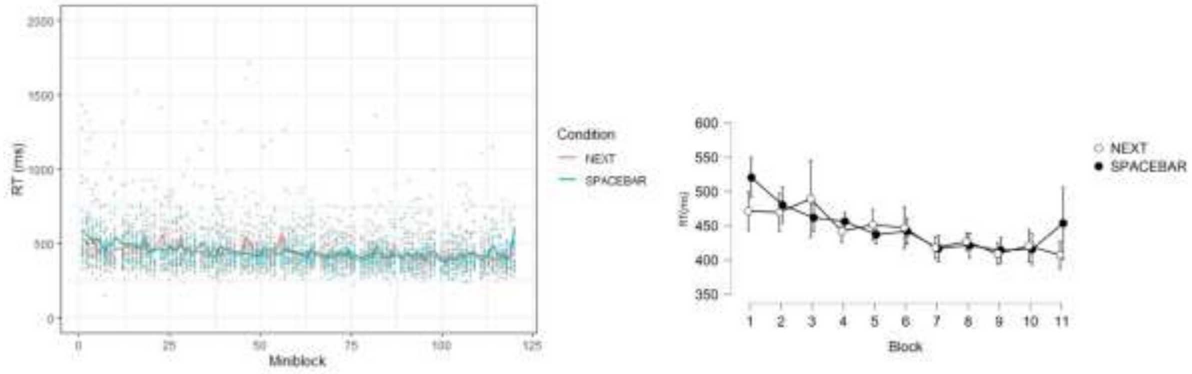

Figure S5. *Left panel: RT in Trial 2 as function of miniblock in Experiment 4, different conditions are marked with different colors. Individual data are shown in dots, and the mean per condition can be seen in the lines. Right panel: RT as a function of Block and Condition, error bars represent 95% Bayesian credible interval*.

Experiment 5

The B/ANOVA showed a robust Block effect [$F(4,156)=6.42$, $p<.001$, $\eta_p^2=0.14$, $BF_{10}=336.07$] without an interaction between Block and Condition, thus accepting the null hypothesis [$F(4,156)=0.27$, $p=.89$, $\eta_p^2<0.01$, $BF_{10}=0.05$].

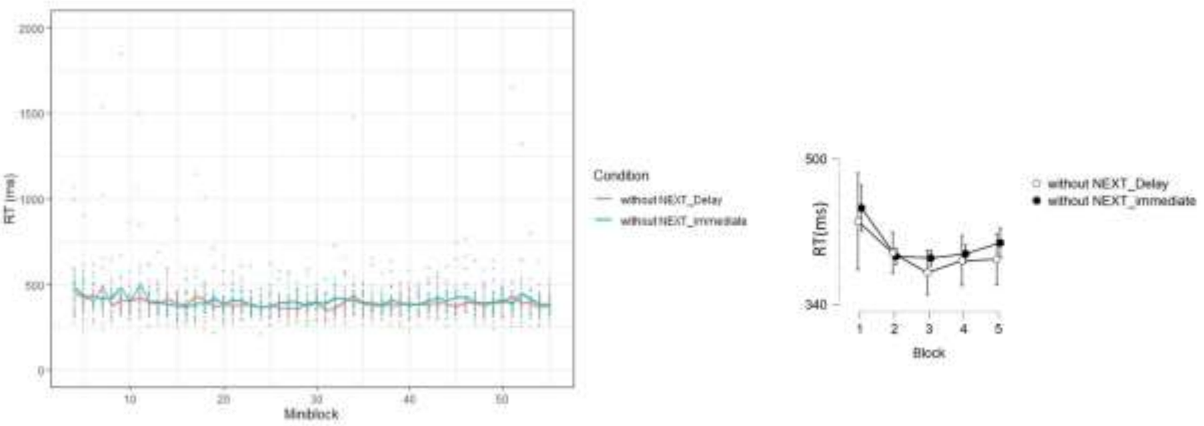

Figure S6. *Left panel: RT in Trial 2 as function of miniblock in Experiment 5, different conditions are marked with different colors. Individual data are shown in dots, and the mean per condition can be seen in the lines. Right panel: RT as a function of Block and Condition, error bars represent 95% Bayesian credible interval*.

References

Jeffreys, H. (1961). *Theory of probability* (3rd ed.). Oxford University Press.

Pashler, H., & Baylis, G. C. (1991). Procedural learning: II Intertrial repetition effects in speeded-choice tasks. *Journal of Experimental Psychology: Learning, Memory, and Cognition*, *17*(1), 33–48. https://doi.org/10.1037/0278-7393.17.1.33
